# The Emerging Role of Immunothrombosis in the Control and Pathogenesis of *Mycobacterium tuberculosis*

**DOI:** 10.1093/infdis/jiaf415

**Published:** 2025-08-05

**Authors:** Seán Donohue, Gina Leisching, Joseph Keane

**Affiliations:** TB Immunology Group, Department of Clinical Medicine, Trinity Translational Medicine Institute, St James's Hospital, Trinity College Dublin, The University of Dublin; Department of Infectious Diseases, St James's Hospital, Dublin, Ireland; TB Immunology Group, Department of Clinical Medicine, Trinity Translational Medicine Institute, St James's Hospital, Trinity College Dublin, The University of Dublin; TB Immunology Group, Department of Clinical Medicine, Trinity Translational Medicine Institute, St James's Hospital, Trinity College Dublin, The University of Dublin

**Keywords:** coagulation, immunothrombosis, thromboinflammation, tissue factor, tuberculosis

## Abstract

Greater understanding of the immunopathogenesis of tuberculosis is critical for developing novel therapies. Here, we propose that immunothrombosis plays an important role in the immune response to *Mycobacterium tuberculosis*. This interplay among macrophages, neutrophils, and platelets leads to microthrombosis at the site of infection, trapping the mycobacterium to prevent dissemination. We explore how dysregulated immunothrombosis might contribute to tuberculosis pathogenesis, with excessive microthrombosis driving drug resistance, leading to lung damage and venous thromboembolism. Further research into these poorly understood mechanisms could identify options for host-directed therapies to ameliorate immunothrombosis, with its attendant tissue destruction, and reduce the burden of resistance.

## Teaser

Accumulating evidence implicates immunothrombosis in the immune response to *Mycobacterium tuberculosis*. Here we outline how its dysregulation may drive thromboembolism, drug resistance, and chronic lung disease, highlighting the need for further study to inform host-directed therapies.

Tuberculosis (TB), caused by *Mycobacterium tuberculosis* (Mtb), remains the biggest infectious killer and 10th-commonest cause of death globally [1]. There are many barriers to its successful elimination, not least of which is multidrug resistance, with an estimated 400 000 cases in 2023 [1]. Even when it is treated successfully, residual chronic lung disease is common [2]. Greater understanding of the immunopathogenic mechanisms that lead to this lung pathology may allow for development of host-directed therapies (HDTs) to ameliorate tissue destruction and improve lung function.

Although the COVID-19 pandemic resulted in an estimated 700 000 excess TB deaths [1], it may have provided clues to better understand the host response to Mtb, particularly relating to immunothrombosis. This process has not previously been linked to TB, but there is growing evidence of its role in the development of lung damage, and it may impair drug delivery to the site of Mtb infection with implications for the development of drug resistance. It is our perspective that further research into this phenomenon may yield targets for HDT to tackle these TB complications.

## IMMUNOTHROMBOSIS

Immunothrombosis is a physiologic process of innate immunity characterized by local formation of microthrombi in response to pathogens, facilitating their recognition, containment, and elimination [3]. This involves activation of coagulation by macrophage- and monocyte-produced tissue factor (TF), neutrophils and their extracellular traps (NETs), and platelets and is described in detail elsewhere [3]. While an important part of innate immunity, it can become dysregulated and contribute to disease pathogenesis as seen with COVID-19 [4].

## THROMBOSIS AND COAGULOPATHY ARE COMMON IN TB

Little is known about the role of immunothrombosis in Mtb response and if its dysregulation contributes to end-organ damage. Although microthrombi were demonstrated postmortem in patients with TB before the availability of antituberculous antimicrobials [5], little emphasis has been placed on them since.

One review suggested that >5% of patients with TB are diagnosed with pulmonary embolism [6], although prevalence varied widely, with higher rates seen in studies that actively screened for venous thromboembolism (VTE) or included more patients who were severely ill or comorbid. A more recent study reported a prevalence of 0.6% [7]. While this is substantially lower, the authors acknowledge that the rate is 6-fold that of the general population. Additionally, certain comorbidities may increase risk, as reflected in the higher prevalence in studies including patients with HIV [6] and by data demonstrating increased coagulation activation and lung damage in patients with diabetes [8].

Coagulopathy is observed in TB disease even without clinically evident thrombosis, suggesting the presence of microthrombosis. Several coagulation factors are elevated in TB, including D-dimer, fibrinogen, and von Willebrand factor (vWF) [9]. Notably, vWF is a marker of endothelial dysfunction, a process central to immunothrombosis in other infections [3, 4]. vWF facilitates platelet-monocyte binding to the endothelium and ultimate thrombus formation [3]. In COVID-19, vWF has been demonstrated to play a role in innate immunity by driving macrophages toward a proinflammatory phenotype [10]. Therefore, the elevated vWF seen in TB may also drive inflammation and indicates how immunothrombosis might contribute to disease severity.

## TF IS UPREGULATED IN TB

TF initiates hemostasis and thrombosis by triggering the extrinsic coagulation pathway, and studies have implicated its role in TB pathogenesis. Mtb-infected human monocyte–derived macrophages have been shown to upregulate TF to a greater and more sustained degree than lipopolysaccharide-treated controls [11]. This points to macrophage-mediated activation of coagulation and a potential mechanism of immunothrombosis in response to Mtb infection.

TF expression by macrophages appears to play a role in controlling Mtb growth. Mice lacking *F3* in their myeloid cells have higher growth of Mtb as compared with wild type mice [12]. TF may also be required for granuloma formation, and transgenic mice with low TF expression form less organized granulomas [13]. Fibrin, a downstream product of TF, is also required for granuloma formation [14]. However, the same study found that fibrinogen-deficient mice had no difference in mycobacterial burden as compared with wild type mice, indicating that these pathways are yet to be fully elucidated. Taken together, we speculate that fibrin may be necessary to provide a physical scaffold for optimal granuloma formation and that its production is mediated by TF-producing macrophages.

Furthermore, a recent proteomic study identified TB-specific enrichment of the intrinsic and common coagulation pathways in the plasma of children when compared with non-TB respiratory disease [15]. Spatial transcriptomics of resected lung tissue of patients with multidrug-resistant TB suggests that activation of coagulation-associated genes may differ across compartments [16]. Downstream of TF, thrombin pathways are most strongly activated in the cavity wall. Equally, there is differential activation of pathways associated with endothelial dysfunction across compartments [16], lending further evidence to the involvement of endothelial activation in this process as outlined here. More recently, a transcriptomics study involving lung tissue from patients post-TB demonstrated that TB drives persistent endothelial dysfunction and thromboinflammatory signaling within the lung vasculature, with these pathways being most upregulated in TB lesions and correlating with those areas with fibrosis [17], implicating immunothrombosis in the development of post-TB lung disease.

## NETS PROMOTE IMMUNOTHROMBOSIS IN TB

NETs participate in immunothrombosis by trapping the microorganism, stimulating the intrinsic coagulation pathway, and increasing platelet aggregation, thus providing a scaffold for microthrombus formation [3]. Entrapment by NETs may have protective effects by allowing macrophages to phagocytose the NET with the trapped Mtb [18]. Conversely, human studies have shown that greater degrees of NET formation correlate with lung damage and cavity formation [19], and their systemic release raises the possibility of their contribution to VTE development in TB [19].

## PLATELETS ARE INVOLVED IN THE INNATE IMMUNE RESPONSE TO MTB AND IMMUNOTHROMBOSIS

The role of platelets in the innate immune response to Mtb has recently been highlighted. Platelet activation markers are elevated in TB disease and return to normal with treatment [20]. Platelets also upregulate matrix metalloproteinase 1 production by monocytes when infected with Mtb, resulting in collagen degradation [20] and potentially lung damage. Moreover, platelets appear to be involved in granuloma formation by facilitating the differentiation of macrophages into multinucleated giant cells [21] but paradoxically may facilitate Mtb replication in granulomas [22]. This again highlights how effector cells of immunothrombosis may be involved in Mtb control and in the immunopathogenesis of TB disease.

## CLINICAL IMPLICATIONS OF IMMUNOTHROMBOSIS IN TB

It seems evident that immunothrombosis is involved in the innate immune response to Mtb, and we hypothesize that it successfully eliminates or contains Mtb in certain individuals early after infection—so-called TB resisters or TB infection, respectively. In the remaining group, immunothrombosis ensues with Mtb multiplication, resulting in its dysregulation, and ultimately contributes to lung damage, reduced blood supply to infected lesions, VTE, and ongoing inflammation as seen in TB disease.

Adequate blood supply is required for antituberculous drugs to reach infected tissue and penetrate the granuloma. Many anti-TB drugs do not penetrate the granuloma adequately and are found at levels associated with the emergence of resistance [23]. Restoring the blood supply to the granuloma may inhibit this.

**Figure 1.**
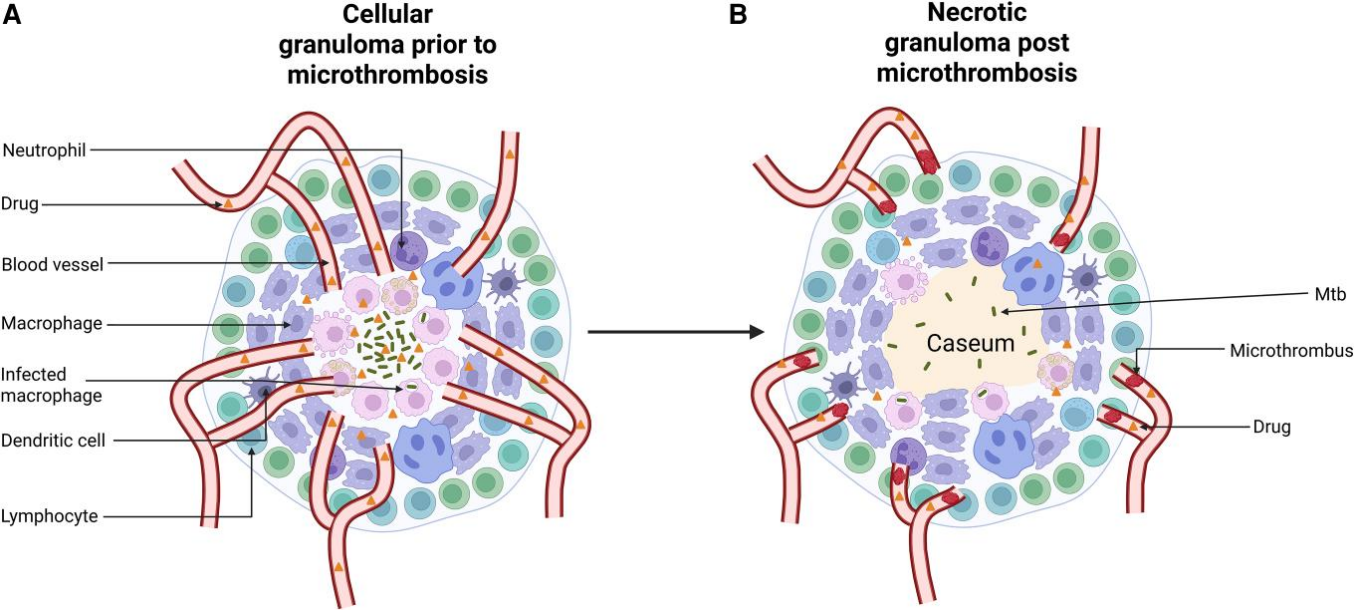
Immunothrombosis may lead to microthrombosis of bloods vessels supplying the granuloma and reduced drug concentration at its center. *A*, Prior to immunothrombosis, the drug is able to reach the center of the cellular granuloma. *B*, As immunothrombosis ensues, microthrombosis obstructs the blood vessels, resulting in reduced drug concentration in the now caseous central granuloma, which may result in the development of drug resistance. Created with BioRender.com.

Alteration and destruction of the vasculature surrounding the granuloma have been demonstrated with micro–computed tomography in lung tissue from patients with TB [24]. We suggest that the microthrombi that form as part of the immune response to Mtb are likely to contribute to this, thus inhibiting drug delivery to the infected lesion via the circulation (Figure 1). As such, it is crucial to elucidate the mechanisms of immunothrombosis in TB so that targets for adjunctive HDT can be identified that may aid in restoring the vasculature to the lesion. Restoring sufficient microcirculation to the granuloma would allow better drug delivery, improve bacillary killing, lower the risk of resistance, and facilitate better immune cell access and oxygenation. In the absence of antituberculous drugs, such a therapy may risk Mtb dissemination. However, with the adjunctive use of this putative HDT with antituberculous antimicrobials, this risk would be mitigated and the potential benefits significant.

Greater understanding of TB immunothrombosis will allow us to better understand the pathways that lead to VTE and chronic lung damage. Much of the data have been provided by in vitro and animal models, and their continued use will elucidate immunothrombotic pathways and identify potential drug targets for study in clinical trials. However, they have obvious limitations and do not fully reflect human Mtb infection. An intriguing and emerging model is that of controlled human TB infection [25], which would allow study of immunothrombotic mechanisms in early human TB.

## CONCLUSION

Several lines of evidence hint at the role of immunothrombosis in the immune response to Mtb and that its dysregulation contributes to pathogenesis. Notwithstanding, there remains a paucity of data on its mechanisms in TB. Further insight could ultimately identify a suitable target for HDT, which may aid in reducing the burden of posttuberculous lung disease and VTE and result in better drug delivery with less emergent drug resistance. More focus on this pathogenic mechanism may represent a significant advancement in current treatment strategies and offer a more effective approach to the management of TB.

## Notes

***Author contributions.*** All authors contributed to the conceptualization and writing of this article.

***Financial support.*** This work was supported by The Royal City of Dublin Hospital Trust.
